# Preclinical Incorporation Dosimetry of [^18^F]FACH—A Novel ^18^F-Labeled MCT1/MCT4 Lactate Transporter Inhibitor for Imaging Cancer Metabolism with PET

**DOI:** 10.3390/molecules25092024

**Published:** 2020-04-26

**Authors:** Bernhard Sattler, Mathias Kranz, Barbara Wenzel, Nalin T. Jain, Rareş-Petru Moldovan, Magali Toussaint, Winnie Deuther-Conrad, Friedrich-Alexander Ludwig, Rodrigo Teodoro, Tatjana Sattler, Masoud Sadeghzadeh, Osama Sabri, Peter Brust

**Affiliations:** 1 Department of Nuclear Medicine, University Hospital Leipzig, 04103 Leipzig, Germany; 2 Helmholtz-Zentrum Dresden-Rossendorf, Institute of Radiopharmaceutical Cancer Research, Department of Neuroradiopharmaceuticals, 04318 Leipzig, Germany; 3 Tromsø PET Center, University Hospital of North Norway, 9009 Tromsø, Norway; 4 Nuclear Medicine and Radiation Biology Research Group, The Arctic University of Norway, 9009 Tromsø, Norway; 5 Department of Claw Animals, University of Leipzig, 04103 Leipzig, Germany

**Keywords:** preclinical radiopharmaceutical dosimetry, image-based internal dosimetry, OLINDA, MCT1/MCT4 lactate transporter inhibitor, [^18^F]FACH, radiation safety

## Abstract

Overexpression of monocarboxylate transporters (MCTs) has been shown for a variety of human cancers (e.g., colon, brain, breast, and kidney) and inhibition resulted in intracellular lactate accumulation, acidosis, and cell death. Thus, MCTs are promising targets to investigate tumor cancer metabolism with positron emission tomography (PET). Here, the organ doses (ODs) and the effective dose (ED) of the first ^18^F-labeled MCT1/MCT4 inhibitor were estimated in juvenile pigs. Whole-body dosimetry was performed in three piglets (age: ~6 weeks, weight: ~13–15 kg). The animals were anesthetized and subjected to sequential hybrid Positron Emission Tomography and Computed Tomography (PET/CT) up to 5 h after an intravenous (iv) injection of 156 ± 54 MBq [^18^F]FACH. All relevant organs were defined by volumes of interest. Exponential curves were fitted to the time–activity data. Time and mass scales were adapted to the human order of magnitude and the ODs calculated using the ICRP 89 adult male phantom with OLINDA 2.1. The ED was calculated using tissue weighting factors as published in Publication 103 of the International Commission of Radiation Protection (ICRP103). The highest organ dose was received by the urinary bladder (62.6 ± 28.9 µSv/MBq), followed by the gall bladder (50.4 ± 37.5 µSv/MBq) and the pancreas (30.5 ± 27.3 µSv/MBq). The highest contribution to the ED was by the urinary bladder (2.5 ± 1.1 µSv/MBq), followed by the red marrow (1.7 ± 0.3 µSv/MBq) and the stomach (1.3 ± 0.4 µSv/MBq). According to this preclinical analysis, the ED to humans is 12.4 µSv/MBq when applying the ICRP103 tissue weighting factors. Taking into account that preclinical dosimetry underestimates the dose to humans by up to 40%, the conversion factor applied for estimation of the ED to humans would rise to 20.6 µSv/MBq. In this case, the ED to humans upon an iv application of ~300 MBq [^18^F]FACH would be about 6.2 mSv. This risk assessment encourages the translation of [^18^F]FACH into clinical study phases and the further investigation of its potential as a clinical tool for cancer imaging with PET.

## 1. Introduction

Aerobic glycolysis is a common feature of cancer physiology. Even under adequate oxygenation, cancer cells generate energy mainly via the cytosolic conversion of glucose into pyruvate and not via the downstream mitochondrial respiratory chain, known as the Warburg effect [1]. Due to the conversion of pyruvate to lactate, this phenomenon results in intracellularly high concentrations of lactate along with a decrease in the pH. Consequently, to avoid apoptosis caused by an acidic environment in the cytoplasm, cancer cells facilitate the proton-coupled efflux of pyruvate and lactate [2,3] by the transmembrane monocarboxylate transporters (MCTs). Upregulation of several MCTs was observed in different cancer types [4,5,6,7,8] and pharmacological inhibition of MCT1/MCT4 impairs cancer cell proliferation and tumor growth [9]. Increased expression of MCT1 and MCT4 in brain malignancies is assumed to be linked to the pathogenesis of glioblastoma in particular [8]. Accordingly, MCT1 and MCT4 are interesting targets for inhibitor-based molecular imaging and pharmacological treatment of this aggressive form of glioma.

Recently, [^18^F]FLac, [^18^F]FP, and an ^11^C-labeled coumarin analog were developed as radiotracers to monitor MCT1 with positron emission tomography (PET). However, defluorination, nonspecific binding, and insufficient affinity hamper the applicability of these tracers [10,11]. Recently, with [^18^F]FACH, a fluorinated analog of α-cyano-4-hydroxycinnamic acid (α-CHC) [6] ((*E*)-2-cyano-3-{4-[(3-[^18^F]fluoropropyl)(propyl)amino]-2-methoxyphenyl}acrylic acid), a novel radiolabeled MCT1/4 inhibitor was developed by our group. The inhibitory potency of FACH (IC_50, MCT1_ = 11 nM; IC_50_, _MCT4_ = 6.5 nM) indicates the suitability of [^18^F]FACH for in vivo molecular imaging of MCT1/MCT4 with PET [12,13].

For the clinical translation of newly developed radiopharmaceuticals, a safety and tolerability assessment is mandatory. One integral part of the assessment is the preclinical radiation dose assessment to estimate the radiation exposure caused by systemic, i.e., intravenous (iv), application of the radiotracer. For the estimation of organ doses (ODs) and the effective dose (ED), the pharmacokinetics and tissue distribution of the tracer have to be determined. The activity concentration in the different organs and tissues is required to determine time-integrated activity concentrations (TIAC) and finally calculate the region-specific numbers of disintegration (NOD). As recently shown, our approach to perform respective PET imaging studies combined with CT or Magnet Resonance Imaging (MRI) in a small number of piglets is suitable to generate pharmacokinetic data for dosimetric calculations [14,15,16,17]. With this fully imaging-based approach [18], the total number of animals used for dosimetry studies can be reduced to a minimum and the acquired data can be re-used for further research questions. It is important to note that when using animals for dose estimation in humans, an interspecies scaling has to be applied [19]. However, as also shown by our previous studies [14,15,16], preclinical dosimetry underestimates the ED in humans by up to 40% independent of the species and size of the test animal, and the radiation dose assessment has to be adapted accordingly.

Herein, we present the results of a preclinical dosimetry study of the newly developed MCT1/MCT4-specific radioligand [^18^F]FACH performed in juvenile pigs with a clinical PET/CT system and report on the estimation of the ED in humans as required for approval of application of the new radiopharmaceutical in clinical studies.

## 2. Results

The radiation safety of the newly developed MCT1/MCT4-specific radioligand [^18^F]FACH was preclinically investigated. Following intravenous injection of 156 ± 54 MBq (0.63 ± 0.49 µg) of the radiopharmaceutical, no adverse effects were observed based on vital sign monitoring, and three piglets were subjected to sequential PET/CT up to 252 min post injection (p.i.). Afterwards, volumes of interest (VOIs) of the various organs were manually defined using the respective whole-body CT dataset for anatomical orientation. The estimated TIACs were extrapolated to the human entity (Equation (2)), followed by estimation of the organ doses with OLINDA 2.1 and calculation of the effective dose using tissue weighting factors as published in Publication 103 of the International Commission of Radiation Protection (ICRP103) [20]. Finally, it can be concluded that [^18^F]FACH is safe with respect to the radiation risk that is caused by its systemic application for PET studies. This supports and encourages the translation of this newly developed radiopharmaceutical to clinical study phases.

**Figure 1:**
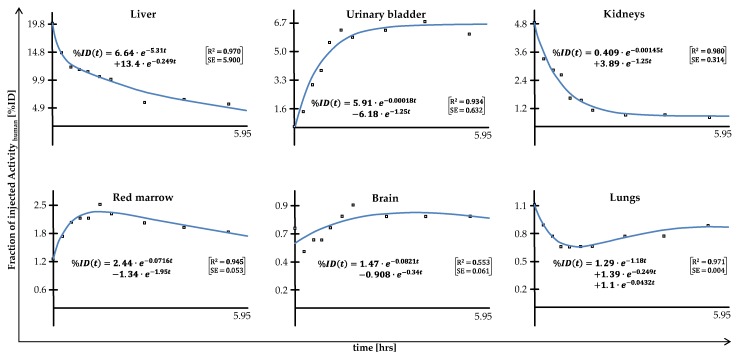
Examples of the mono-, bi-, or tri-exponential fits, including exponential fit equations and parameters of fit goodness (R-squared and squared error) of the human scale time–activity data using the EXM-Module of OLINDA. All fits are presented in the Supplementary Materials (Figures S1–S3).

The TIAC courses in organs allow for determination of the fraction of administered activity, also referred to as percent of injected dose (%ID), in the particular region, followed by calculation of the NOD by integration over time applied at mono-, bi-, or tri-exponentially fitted TIACs. Figure 1 shows six examples of the exponential fits of the fractions of activity (%ID) in different organs over time (all scaled to human dimensions). The fits of all organs of the three investigated animals as well as the exact exponential equations of the fits can be found in the Supplementary Materials (Figures S1–S3).

**Figure 2:**
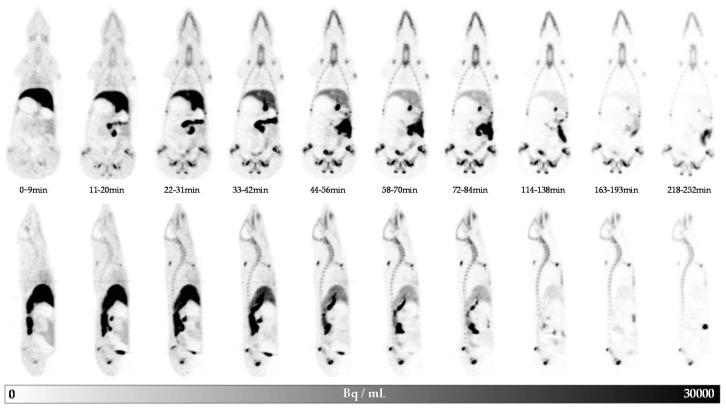
Whole body dynamic positron emission tomography (PET) images in coronal (upper row) and sagittal (lower row) views of pig 1 after intravenous (iv) application of 191 MBq [^18^F]FACH.

In Figure 2, the distribution of activity in one representative animal at different times p.i. of [^18^F]FACH is presented, showing a high uptake in the liver, pancreas, small intestines, and spleen. The rapid uptake in the liver and later also in the gall bladder indicates hepatobiliary excretion. Early accumulation in the bone structures might be related to defluorination. At later time points, activity also accumulated in the gall bladder and the urinary bladder.

**Figure 3:**
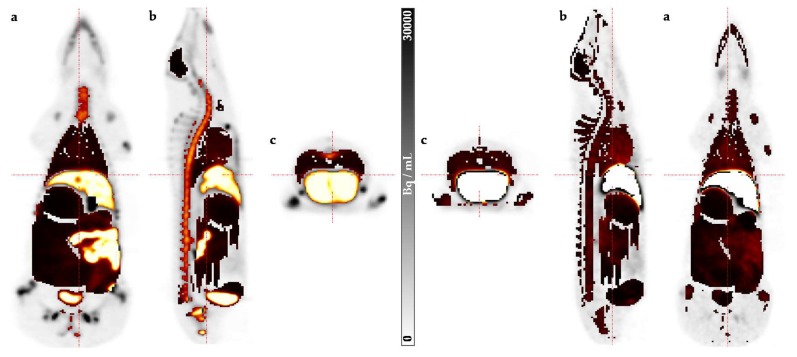
PET image with volumes of interest (VOIs) superimposed in (**a**) coronal, (**b**) sagittal, and (**c**) transversal views. The left panel, pig 1, obtained at 42 min post injection (p.i.), shows the organs except for the peripheral bone, whereas the right panel, pig 2, obtained at 56 min. p.i., shows all delineated organs. (The gray scale in the middle refers to the original quantitative PET data, not to the colored VOI structures).

An example of manually delineated VOIs using the PET or CT information to extract the accumulated amount of activity in the respective organ is presented in Figure 3. All biodistribution data, including TIACs for all organs, are available in the Supplementary Materials (Tables S1–S4; Figure S1).

Based on the biokinetic data extracted from the VOI analysis in piglets and the following extrapolation to the human entity (Equations (3) and (4)), the ODs and the ED were estimated for the adult male model. The resulting mean values obtained for 24 organs are presented in Table 1.

Here, the effective dose (*E*) represents the tissue-weighted sum of the equivalent doses in all specified tissues and organs of the body (Equation (1)), where *H_T_* is the equivalent dose in the respective tissue or organ, *T*, and *w_T_* is the tissue weighting factor [20].

(1) $$E=\underset{T}{\sum }w_{T}\times H_{T}\left[\frac{mSv}{MBq}\right]$$

The highest OD was received by the urinary bladder (62.6 ± 28.9 µSv/MBq), followed by the gall bladder (50.4 ± 37.5 µSv/MBq), the pancreas (30.5 ± 27.3 µSv/MBq), the kidneys (26.2 ± 2.2 µSv/MBq), the right colon (24.0 ± 7.4 µSv/MBq), and the liver (23.3 ± 13.6 µSv/MBq). When involving the tissue weighting factor *w_T_*, which weights the organ equivalent dose in a tissue or organ to represent the relative contribution of that tissue or organ to the total health risk resulting from uniform irradiation of the body, this ranking changes: the highest contribution to the ED is by the urinary bladder (2.5 ± 1.1 µSv/MBq), followed by the red marrow (1.7 ± 0.3 µSv/MBq), the stomach (1.3 ± 0.4 µSv/MBq), the right colon (1.2 ± 0.4 µSv/MBq), the lungs (0.9 ± 0.05 µSv/MBq), and the liver (0.1 ± 0.05 µSv/MBq). The ED was found to be 12.4 µSv/MBq. According to this preclinically obtained data, a standard injection in humans of 300 MBq [^18^F]FACH for PET in three-dimensional (3D) mode would result in an ED of about 3.7 mSv. As known from other studies [21], preclinical incorporation dose estimates underrate the ED to humans by up to 40% [14,15,16]. Taking this into account, the conversion factor to estimate the ED to humans undergoing a clinical PET investigation using [^18^F]FACH would rise to 20.6 µSv/MBq and finally result in an ED of about 6.2 mSv/300 MBq of administered [^18^F]FACH. In comparison to ED values determined for other clinically applied diagnostic radiotracers [21], this risk assessment encourages the translation of [^18^F]FACH into clinical study phases and the further investigation of its potential as a clinical tool for PET imaging of MCT1/MCT4 in different pathologies, including oncological diseases.

## 3. Discussion

Due to metabolic reprogramming, highly proliferating cancer cells utilize large amounts of glucose and convert the glucose carbon mainly to lactate to support their anabolic requirements [22,23,24]. Given the availability of suitable radiolabeled agents, non-invasive molecular imaging by PET enables the investigation of cancer-related alterations in the metabolism of cells. For example, the enhanced uptake of glucose in cancer cells can be monitored by [^18^F]fluoro-deoxyglucose ([^18^F]FDG), a substrate of the glucose transporters (GLUT) overexpressed in cancer cells that has been clinically used for more than three decades for diagnosis, staging, and treatment monitoring in oncology [25,26]. Monocarboxylate transporters (MCTs), which mediate the proton-coupled transport of small carboxylic acids such as lactate, the end product of aerobic glycolysis, are also known to be overexpressed in different cancers [27,28]. Accordingly, ^11^C- or ^18^F-labeled substrates of MCTs such as [^18^F]lactate and [^11^C]pyruvate have already been developed and evaluated regarding their potential to image the expression of MCTs in tumors using PET [10,29,30]. Besides, highly affine and small molecule inhibitors of MCTs designed for targeted cancer therapies also bear the potential for the development of respective imaging agents. In this context, our group has recently reported on the development and evaluation of [^18^F]FACH as a new MCT-targeting imaging agent possessing high inhibitory potency towards MCT1 and MCT4 [12,13]. For further assessment of the suitability of this radiopharmaceutical in clinical settings, a preclinical dosimetry study is necessary to estimate the doses delivered to humans to ensure the safe usage of [^18^F]FACH.

According to the data obtained in this study, the overall dose estimate and vital signs monitoring confirm the radiation safety and tolerability of [^18^F]FACH and support the translation of [^18^F]FACH to further clinical study phases.

As found for other low-molecular-weight ^18^F-labeled tracers, which were investigated by our group in recent years (Table 2), the organs involved in the renal as well as the hepatobiliary excretion received the highest ODs. Due to the high initial uptake of activity in the liver, although decreasing over time, the surrounding tissues, such as lung, pancreas, and kidney tissues, are exposed to comparatively high doses and thus belong to the organs contributing mainly to the ED. Furthermore, the increasing concentration in urine results in the high OD and subsequently ED of the urinary bladder wall. Notably, besides being excretory organs, the liver and kidneys are also target organs due to a high expression of MCT1/MCT4 [31,32,33,34,35].

A limitation of this preclinical study is that the piglets did not void during the entire imaging phase, which results in a simplification of the data analysis. As the voiding bladder model implemented in the OLINDA software cannot be applied, the dose estimation for the wall of the urinary bladder is purely imaging based and uses data based on a continuously filling bladder over the imaging time (see Figure 1). Therefore, to reduce the dose to this dose-limiting organ in humans, participants of clinical studies should be instructed to void the bladder before and immediately after each imaging session.

Another limitation of this study is related to the increasing uptake of activity in the bones. Anatomically, high amounts of the red marrow are found not only in the backbone, sternum, and pelvis but also in the epiphyseal plates of the peripheral bone of juvenile piglets, because bone growth is not yet complete. Hence, target-specific accumulation of activity due to physiological expression of MCTs in erythrocytes produced by the red bone marrow [36,37] may contribute to comparatively high ODs and finally explain the high ED contribution of bone marrow. In addition, accumulation of [^18^F]fluoride is indicated by the radio-chromatographic analysis of plasma samples obtained during the PET imaging study (data are not shown).

Furthermore, a limitation of preclinical dosimetry studies is that the animals usually have to be anesthetized. Depending on the target of the tracer under investigation, anesthesia will have an influence on biodistribution and biokinetics. In this study, however, the anesthesia should have had a rather secondary influence. First and foremost, the anesthesia as described does not affect MCT1/MCT4 transporter inhibitors. It does act in the central nervous system (mainly the brain). With our protocol, we maintained a rather shallow anesthesia so that the animals respired spontaneously. Thus, the alterations of the biodistribution and biokinetics of [^18^F]FACH by this anesthesia should have had a rather small influence on the dosimetry result. We expect a slight general deceleration of the metabolism, particularly that of the gastro-intestinal tract, just as while sleeping very deeply. However, animal anesthesia is generally necessary to render preclinical incorporation dosimetry possible. Although it is a well-known limitation of preclinical dosimetry studies, it does not invalidate their results. Moreover, alongside the other limitations of preclinical dosimetry described, it will be part of the comparison once clinical data have been acquired and be involved in the (up)scaling of preclinical ED results for the assessment of the ED to humans by a particular radiotracer.

## 4. Materials and Methods

### 4.1. Synthesis of [^18^F]FACH

**Figure 4:**
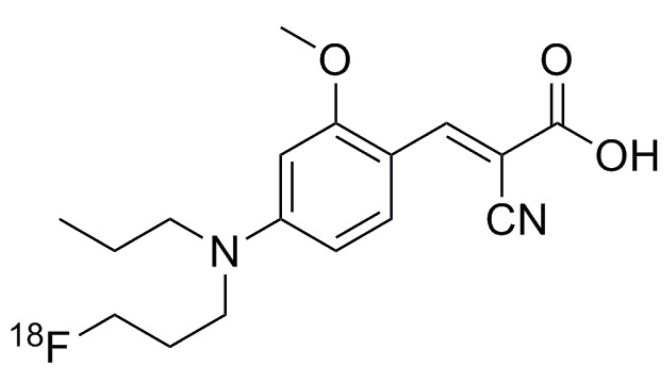
Chemical structure of [^18^F]FACH.

The radiosynthesis of [^18^F]FACH has recently been published [38]. Briefly, [^18^F]FACH is produced via a one-step radiosynthesis approach by using a mesylate precursor bearing an unprotected carboxylic acid function and the Kryptofix 2.2.2/K_2_CO_3_/[^18^F]F-complex system. Isolation of [^18^F]FACH was performed by semi-preparative HPLC (Reprosil-Pur C18-AQ column, 250 × 10 mm, 10 µm, 50% CH_3_CN/20 mM NH_4_HCO_2_ (aq.), pH = 4–4.5, flow 3.5 mL/min). The tracer (chemical structure shown in Figure 4) was finally purified via solid-phase extraction (Sep-Pak^®^ C18 light cartridge) and formulated in 10% EtOH/saline solution with molar activities in the range of 50–120 GBq/µmol (*n* = 8, at the end of synthesis) using starting activities of 1–3 GBq.

### 4.2. Preclinical Dosimetry Studies—In Vivo PET/CT Imaging in Pigs

All animal experiments were approved by the responsible institutional and federal state authorities (Landesdirektion Leipzig; TVV 18/18, Reference Number DD24.1-5131/446/19).

**Figure 5:**
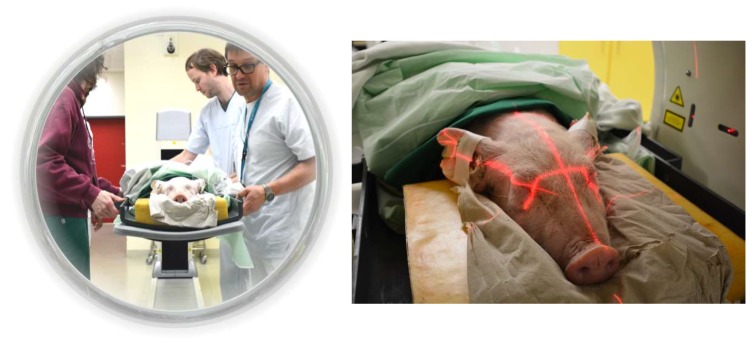
The animals were placed in a plastic trough and a special head rest to guarantee reproducible positioning and avoid the movement of artefacts.

**Figure 6:**
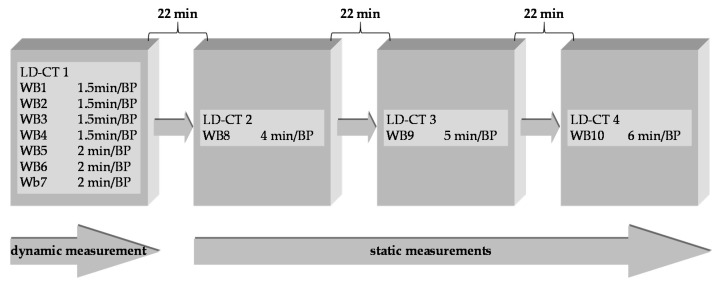
PET/CT imaging protocol comprising a dynamic and static part with increasing duration per bed position (BP) to compensate for decay and, thus, decreasing count statistics, preceded by a low-dose CT (LD-CT) for attenuation correction and anatomical orientation, respectively. Positioning of the animal in the PET field of view.

Three piglets (age: ~6 weeks, weight: ~13–15 kg) were fasted on the day of imaging and received an intranuscular. injection of 1 mL azaperone and 4 mL ketamine to introduce anesthesia. After 15 min, 2 mL of ketamine and 1 mL of midazolam (5 mg/mL) were iv injected (ear vein, V. auricularis), followed by 5 mL of G40, 3 mL of ketamine, and 1.5 mL of midazolam in 50 mL of NaCl 0.9% with an infusion pump at a flow rate of 37.5 mL/h to maintain the narcosis throughout the entire investigation time. During narcosis, the animals maintained spontaneous respiration and no mandatory ventilation was applied. The subjects were sequentially imaged after an iv injection (contralateral ear vein) of 156 ± 54 MBq [^18^F]FACH (0.63 ± 0.49 µg) in a PET/CT system (Biograph16, SIEMENS, Erlangen, Germany). The piglets were positioned prone with legs alongside the body on a custom-made plastic trough including a piglet head-holder (Figure 5). The PET acquisition was divided into a sequential (4 × 9 min, 3 × 12 min) and a static part (1 × 24 min, 1 × 30 min, 1 × 36 min), each of which was preceded by a low-dose CT to acquire structural data for attenuation correction (AC) and anatomical orientation (Figure 6). Post mortem, the urine was collected by bladder punctuation, weighted, and divided into three 1 mL samples for activity measurements in a gamma-counter (Packard Cobra II 5003 Auto Gamma Counting System, GMI, Ramsey, MN, USA).

PET data reconstruction was done using low-dose CT attenuation correction (AC) and an iterative OSEM algorithm with 4 iterations and 8 subsets. As the PET/CT system is in daily clinical use, it is periodically subjected to detector normalization and activity calibration. Furthermore, all peripheral activity-measuring devices to be used for the investigation (dose calibrator, gamma counter) are cross-calibrated in terms of timing and a radioactivity adjustment.

### 4.3. Image Analysis

The image data were analyzed with ROVER (ABX, Radeberg, Germany; v. 3.0.46h). The organs were identified and manually delineated using 3D volumes of interest (VOI). The CT data were used for anatomical orientation and for image registration with the PET data. Relevant source organs like brain, gall bladder, large intestine, small intestine, stomach, heart, kidneys, liver, lungs, pancreas, red marrow (backbone, pelvis, sternum), spleen, thyroid, testes, skeleton (bone), and the urinary bladder were delineated and the time–activity data transformed into percentage of injected dose (%*ID_organ_*) with Equation (2).

(2) $${\%ID_{organ}}_{t}=\frac{{A_{organ}}_{t}\times {c_{scan}}_{t}}{{A_{0}}_{t}}\;\left[\%\right]$$

where ${A_{organ}}_{t}$ is the activity in the organ at the time *t*; ${c_{scan}}_{t}$ is a calibration factor representing the theoretical body activity (derived from a whole body mask of a volume that equals the body dimentions of the animal) decay corrected to *t* and divided by the imaged body activity in each image frame at the time *t*, and ${A_{0}}_{t}$ is the injected activity decay corrected to *t*_0_.

### 4.4. Incorporation Dosimetry

The dose calculation has been described by our group in detail before [14,15,16]. Briefly, due to differences in weight, size, and metabolic rates between the animal species and the human volunteers, it is necessary to map the preclinical extracted biodistribution data to human circumstances. Thus, the animal biokinetic data (time scale and *%ID* values) were adapted to the human circumstances according to [22] to fit the human weight, size, and metabolic rates (Equations (3) and (4)).

(3) $$t_{\mathrm{human}}=t_{\mathrm{animal}}{\left[\frac{m_{\mathrm{human}}}{m_{\mathrm{animal}}}\right]}^{0.25}$$

(4) $$[\%\mathrm{ID}]/{\mathrm{organ}}_{\mathrm{human}}=[\%\mathrm{ID}]/g_{\mathrm{animal}}\times \frac{m_{{\mathrm{TB}}_{\mathrm{animal}}}\left[g\right]}{m_{{\mathrm{TB}}_{\mathrm{human}}}\left[g\right]}{\times m}_{{\mathrm{organ}}_{\mathrm{human}}}\left[g\right]$$

The TIACs were estimated by exponential fitting and the dosimetry estimation was performed using OLINDA/EXM software (v. 2.1). Finally, organ doses (OD) were estimated and multiplied by tissue weighting factors as published in ICRP103 [20].

## 5. Conclusions

By extrapolation of the preclinical dosimetry data obtained in piglets, the effective dose as a measure of the overall radiation risk upon iv application of about 300 MBq [^18^F]FACH to humans is estimated to be 3.7 mSv. However, as frequently observed for other PET tracers, the preclinical dosimetry underestimates the ED to humans by up to 40%. Accordingly, we conclude that the ED to humans by [^18^F]FACH can be expected to be about 20.6 µSv/MBq. With a systemic application of 300 MBq, PET imaging with [^18^F]FACH would yield an effective dose of about 6.2 mSv to human subjects. This value is well within the range of other ^18^F-labeled radiopharmaceuticals. Despite the study-specific overestimation of the contributions of bone and the urinary bladder to the ED, this risk assessment encourages the transference of [^18^F]FACH from preclinical to clinical study phases to further assess the suitability of this new radiopharmaceutical for PET imaging of oncological diseases.

## Figures and Tables

**Table 1:** Complete results of the dose assessment of the three investigated piglets using OLINDA 2.1 (mean value ± standard deviation (SD)).

Target Organ	OD	ED Contr.	ED Contr.	SD
	in mSv/MBq	in ± mSv/MBq	in mSv/MBq	in ± mSv/MBq
Adrenals	1.42 × 10^−2^	5.69 × 10^−4^	1.31 × 10^−4^	4.73 × 10^−6^
Brain	4.69 × 10^−3^	8.09 × 10^−4^	4.69 × 10^−5^	8.09 × 10^−6^
Esophagus	9.03 × 10^−3^	1.21 × 10^−3^	3.61 × 10^−4^	4.86 × 10^−5^
Eyes	6.91 × 10^−3^	1.68 × 10^−3^	0.00	0.00
Gall Bladder Wall	5.04 × 10^−2^	3.75 × 10^−2^	4.65 × 10^−4^	3.47 × 10^−4^
Left Colon	1.37 × 10^−2^	2.91 × 10^−3^	6.63 × 10^−4^	1.41 × 10^−4^
Small Intestine	1.84 × 10^−2^	8.44 × 10^−3^	1.71 × 10^−4^	7.81 × 10^−5^
Stomach Wall	1.09 × 10^−2^	3.07 × 10^−3^	1.31 × 10^−3^	3.64 × 10^−4^
Right Colon	2.40 × 10^−2^	7.40 × 10^−3^	1.16 × 10^−3^	3.62 × 10^−4^
Rectum	1.38 × 10^−2^	1.15 × 10^−3^	3.18 × 10^−4^	2.60 × 10^−5^
Heart Wall	9.46× 10^−3^	7.51 × 10^−4^	8.72 × 10^−5^	6.72 × 10^−6^
Kidneys	2.62 × 10^−2^	2.19 × 10^−3^	2.42 × 10^−4^	2.00 × 10^−5^
Liver	2.33 × 10^−2^	1.36 × 10^−2^	9.34 × 10^−4^	5.46 × 10^−4^
Lungs	7.88 × 10^−3^	2.27 × 10^−4^	9.46 × 10^−4^	2.72 × 10^−5^
Pancreas	3.05 × 10^−2^	2.73 × 10^−2^	2.81 × 10^−4^	2.52 × 10^−4^
Prostate	1.30 × 10^−2^	2.85 × 10^−3^	6.01 × 10^−5^	1.32 × 10^−5^
Salivary Glands	7.90 × 10^−3^	2.11 × 10^−3^	7.90 × 10^−5^	2.11 × 10^−5^
Red Marrow	1.39 × 10^−2^	2.19 × 10^−3^	1.67 × 10^−3^	2.63 × 10^−4^
Osteogenic Cells	2.12 × 10^−2^	1.88 × 10^−3^	2.12 × 10^−4^	1.88 × 10^−5^
Spleen	9.08 × 10^−3^	1.14 × 10^−3^	8.37 × 10^−5^	1.04 × 10^−5^
Testes	8.45 × 10^−3^	2.21 × 10^−3^	3.38 × 10^−4^	8.87 × 10^−5^
Thymus	8.33 × 10^−3^	1.73 × 10^−3^	7.69 × 10^−5^	1.59 × 10^−5^
Thyroid	7.08 × 10^−3^	8.70 × 10^−4^	2.83 × 10^−4^	3.49 × 10^−5^
Urinary Bladder Wall	6.26 × 10^−2^	2.89 × 10^−2^	2.50 × 10^−3^	1.15 × 10^−3^
Total Body	9.76 × 10^−3^	1.53 × 10^−3^	0.00	0.00
ED			**12.4 × 10^−2^**	

OD = organ equivalent dose; ED contr. = effective dose contribution.

**Table 2:** Dosimetry results of different PET radiopharmaceuticals preclinically estimated using piglet biokinetic data.

Tracer	Target/Organ	Preclinical (μSv/MBq)	Reference
[^18^F]FACH	MCT1/4/brain tumor	12.4	this study
[^18^F]DBT10	α7 receptor/brain tumor	13.7	[22]
(S)-(−)-[^18^F]fluspidine (R)-(+)-[^18^F]fluspidine	σ receptor/brain	12.9 14.0	[15]
(−)-[^18^F]flubatine (+)-[^18^F]flubatine	α4β2 receptor/brain	14.7 14.3	[16] [14]

## Supplementary Materials

The following data are available online: Tables S1–S3: %ID values of the three piglets after i.v. injection of [^18^F]FACH. Table S4: Detailed results of the dose calculation for the three animals: organ equivalent doses and effective dose contributions involving the tissue risk factor wT of ICRP103. Figures S1–S3: All time-integrated activity curves with fitting functions and fit goodness parameters (R-squared and squared error) of all organs and systems of organs that could be identified as taking up the tracer for all included animals.

## References

[1] Warburg O. (1956). On the origin of cancer cells. Science.

[2] Ponisovskiy M.R. (2011). Warburg effect mechanism as the target for theoretical substantiation of a new potential cancer treatment. Crit. Rev. Eukaryot. Gene Expr..

[3] Koppenol W.H., Bounds P.L., Dang C.V. (2011). Otto Warburg’s contributions to current concepts of cancer metabolism. Nat. Rev. Cancer.

[4] Halestrap A.P. (2013). The SLC16 gene family–structure, role and regulation in health and disease. Mol. Asp. Med..

[5] Pinheiro C., Longatto-Filho A., Azevedo-Silva J., Casal M., Schmitt F.C., Baltazar F. (2012). Role of monocarboxylate transporters in human cancers: State of the art. J. Bioenerg. Biomembr..

[6] Gurrapu S., Jonnalagadda S.K., Alam M.A., Nelson G.L., Sneve M.G., Drewes L.R., Mereddy V.R. (2015). Monocarboxylate transporter 1 inhibitors as potential anticancer agents. ACS Med. Chem. Lett..

[7] Fang J., Quinones Q.J., Holman T.L., Morowitz M.J., Wang Q., Zhao H., Sivo F., Maris J.M., Wahl M.L. (2006). The H+-linked monocarboxylate transporter (MCT1/SLC16A1): A potential therapeutic target for high-risk neuroblastoma. Mol. Pharmacol..

[8] Park S.J., Smith C.P., Wilbur R.R., Cain C.P., Kallu S.R., Valasapalli S., Sahoo A., Guda M.R., Tsung A.J., Velpula K.K. (2018). An overview of MCT1 and MCT4 in GBM: Small molecule transporters with large implications. Am. J. Cancer Res..

[9] Payen V.L., Mina E., van Hée V.F., Porporato P.E., Sonveaux P. (2020). Monocarboxylate transporters in cancer. Mol. Metab..

[10] van Hée V.F., Labar D., Dehon G., Grasso D., Grégoire V., Muccioli G.G., Frédérick R., Sonveaux P. (2017). Radiosynthesis and validation of (±)-[^18^F]-3-fluoro-2-hydroxypropionate ([^18^F]-FLac) as a PET tracer of lactate to monitor MCT1-dependent lactate uptake in tumors. Oncotarget.

[11] Tateishi H., Tsuji A.B., Kato K., Sudo H., Sugyo A., Hanakawa T., Zhang M.-R., Saga T., Arano Y., Higashi T. (2017). Synthesis and evaluation of ^11^C-labeled coumarin analog as an imaging probe for detecting monocarboxylate transporters expression. Bioorganic Med. Chem. Lett..

[12] Sadeghzadeh M., Moldovan R.-P., Fischer S., Wenzel B., Ludwig F.-A., Teodoro R., Deuther-Conrad W., Jonnalagadda S., Jonnalagadda S.K., Gudelis E. (2019). Development and radiosynthesis of the first ^18^F-labeled inhibitor of monocarboxylate transporters (MCTs). J. Label. Comp. Radiopharm..

[13] Sadeghzadeh M., Moldovan R.-P., Wenzel B., Kranz M., Deuther-Conrad W., Toussaint M., Fischer S., Ludwig F.-A., Teodoro R., Jonnalagadda S.K. (2019). Development of the first ^18^F-labeled MCT1/MCT4 lactate transport inhibitor: Radiosynthesis and preliminary in vivo evaluation in mice. J. Label. Comp. Radiopharm..

[14] Kranz M., Sattler B., Tiepolt S., Wilke S., Deuther-Conrad W., Donat C.K., Fischer S., Patt M., Schildan A., Patt J. (2016). Radiation dosimetry of the α 4 β 2 nicotinic receptor ligand (+)-[^18^F] flubatine, comparing preclinical PET/MRI and PET/CT to first-in-human PET/CT results. EJNMMI Phys..

[15] Kranz M., Sattler B., Wüst N., Deuther-Conrad W., Patt M., Meyer P., Fischer S., Donat C., Wünsch B., Hesse S. (2016). Evaluation of the enantiomer specific biokinetics and radiation doses of [^18^F] fluspidine—A new tracer in clinical translation for imaging of σ1 receptors. Molecules.

[16] Sattler B., Kranz M., Starke A., Wilke S., Donat C.K., Deuther-Conrad W., Patt M., Schildan A., Patt J., Smits R. (2014). Internal Dose assessment of (–)-^18^F-flubatine, comparing animal model datasets of mice and piglets with first-in-human results. J. Nucl. Med..

[17] Stabin M.G., Siegel J.A. (2018). RADAR dose estimate report: A compendium of radiopharmaceutical dose estimates based on OLINDA/EXM version 2.0. J. Nucl. Med..

[18] McParland B.J. (2010). Nuclear Medicine Radiation Dosimetry: Advanced Theoretical Principles.

[19] Stabin M.G. (2008). Fundamentals of Nuclear Medicine Dosimetry.

[20] Valentin J. (2007). The 2007 Recommendations of the International Commission on Radiological Protection.

[21] Zanotti-Fregonara P., Lammertsma A.A., Innis R.B. (2013). Suggested pathway to assess radiation safety of 18 F-labeled PET tracers for first-in-human studies. Eur. J. Nucl. Med. Mol. Imaging.

[22] Kranz M., Sattler B., Deuther-Conrad W., Teodoro R., Donat C., Wenzel B., Scheunemann M., Patt M., Sabri O., Brust P. (2014). Preclinical dose assessment and biodistribution of [^18^F]DBT10, a new α7 nicotinic acetylcholine receptor (α7-nAChR) imaging ligand. J. Nucl. Med..

[23] Tennant D.A., Durán R.V., Gottlieb E. (2010). Targeting metabolic transformation for cancer therapy. Nat. Rev. Cancer.

[24] Ward P.S., Thompson C.B. (2012). Metabolic reprogramming: A cancer hallmark even warburg did not anticipate. Cancer Cell.

[25] O’Neill H., Malik V., Johnston C., Reynolds J.V., O’Sullivan J. (2019). Can the efficacy of ^18^FFDG-PET/CT in clinical oncology be enhanced by screening biomolecular profiles?. Pharmaceuticals (Basel).

[26] Endo K., Oriuchi N., Higuchi T., Iida Y., Hanaoka H., Miyakubo M., Ishikita T., Koyama K. (2006). PET and PET/CT using ^18^F-FDG in the diagnosis and management of cancer patients. Int. J. Clin. Oncol..

[27] Payen V.L., Hsu M.Y., Rädecke K.S., Wyart E., Vazeille T., Bouzin C., Porporato P.E., Sonveaux P. (2017). Monocarboxylate transporter MCT1 promotes tumor metastasis independently of its activity as a lactate transporter. Cancer Res..

[28] Jones R.S., Morris M.E. (2016). Monocarboxylate transporters: Therapeutic targets and prognostic factors in disease. Clin. Pharmacol. Ther..

[29] Herrero P., Dence C.S., Coggan A.R., Kisrieva-Ware Z., Eisenbeis P., Gropler R.J. (2007). L-3-^11^C-lactate as a PET tracer of myocardial lactate metabolism: A feasibility study. J. Nucl. Med..

[30] Yokoi F., Hara T., Iio M., Nonaka I., Satoyoshi E. (1990). 1-^11^Cpyruvate turnover in brain and muscle of patients with mitochondrial encephalomyopathy. A study with positron emission tomography (PET). J. Neurol. Sci..

[31] Koho N., Maijala V., Norberg H., Nieminen M., Pösö A.R. (2005). Expression of MCT1, MCT2 and MCT4 in the rumen, small intestine and liver of reindeer (Rangifer tarandus tarandus L.). Comp. Biochem. Physiol. Part A Mol. Integr. Physiol..

[32] de Araujo G.G., Gobatto C.A., de Barros Manchado-Gobatto F., Teixeira L.F., Dos Reis I.G., Caperuto L.C., Papoti M., Bordin S., Cavaglieri C.R., Verlengia R. (2015). MCT1 and MCT4 kinetic of mRNA expression in different tissues after aerobic exercise at maximal lactate steady state workload. Physiol. Res..

[33] Sepponen K., Ruusunen M., Pakkanen J.A., Pösö A.R. (2007). Expression of CD147 and monocarboxylate transporters MCT1, MCT2 and MCT4 in porcine small intestine and colon. Vet. J..

[34] Becker H.M., Mohebbi N., Perna A., Ganapathy V., Capasso G., Wagner C.A. (2010). Localization of members of MCT monocarboxylate transporter family Slc16 in the kidney and regulation during metabolic acidosis. Am. J. Physiol. Renal Physiol..

[35] Wang Q., Lu Y., Yuan M., Darling I.M., Repasky E.A., Morris M.E. (2006). Characterization of monocarboxylate transport in human kidney HK-2 cells. Mol. Pharm..

[36] Deuticke B. (1982). Monocarboxylate transport in erythrocytes. J. Membr. Biol..

[37] Poole R.C., Cranmer S.L., Halestrap A.P., Levi A.J. (1990). Substrate and inhibitor specificity of monocarboxylate transport into heart cells and erythrocytes. Further evidence for the existence of two distinct carriers. Biochem. J..

[38] Sadeghzadeh M., Moldovan R.-P., Teodoro R., Brust P., Wenzel B. (2019). One-step radiosynthesis of the MCTs imaging agent [^18^F]FACH by aliphatic ^18^F-labelling of a methylsulfonate precursor containing an unprotected carboxylic acid group. Sci. Rep..

