# Supplementary material for: Preclinical Incorporation Dosimetry of [18F]FACH—A Novel 18F-Labeled MCT1/MCT4 Lactate Transporter Inhibitor for Imaging Cancer Metabolism with PET
Source: Molecules. 2020 Apr 26;25(9):2024. doi: 10.3390/molecules25092024 (PMC7248880; doi:10.3390/molecules25092024)
Supplement: Supplementary file 1 [file molecules-25-02024-s001.pdf]

Supplementary Materials

Preclinical Incorporation Dosimetry of [^{18}F]FACH—A Novel ^{18}F-Labeled MCT1/MCT4 Lactate Transporter Inhibitor for Imaging Cancer Metabolism with PET

Bernhard Sattler, Mathias Kranz, Barbara Wenzel, Nalin T. Jain, Rareş-P. Moldovan, Magali Toussaint, Winnie Deuther-Conrad, Alexander F. Ludwig, Rodrigo Teodoro, Tatjana Sattler, Masoud Sadeghzadeh, Osama Sabri and Peter Brust

Affiliations and contributions of the authors are listed in the main manuscript.

Supplemental Methods and Results

In this supplemental material we present the complete time-activity data of all subjects (Table S1 to S3). Moreover, we show the time integrated activity fit functions that the numbers of disintegrations (NODs) for each identifiable organ and system of organs were integrated from. Some singe regions were occasionally not identifiable, neither in the structural CT data nor by considerable uptake of the tracer. This happens particularly in very small regions or if their uptake is comparatively low. This situation is marked in the tables as well as in the fit charts.

Another situation that is obvious from the regions that we delineated is that those stem from the legacy ORNL phantom. On the other hand, the dose tables show organs and systems of organs of the ICRP89 adult male phantom. This is explained by the fact that – due to limitations of local availability – we still used OLINDA/EXM 1.1 for fitting the regions resulting in NODs for the legacy ORNL adult male phantom. Using ICRP 103 tissue weighing factors requires dose calculations using the ICRP 89 phantoms. We adapted the NODs for those phantoms. Were possible we used the NOD-data 1:1. In the gut region that has been re-organized to be split into right and left colon and the rectum in the ICRP 89 phantoms, the adaption was done so that the upper large intestine (ULI) region of the ORNL-phantom was set equal to the right colon of the ICRP 89 phantom and the lower large intestine of the legacy phantom was split half and half representing the Left colon and the rectum. As this is no exact anatomical match it represents some kind of a limitation of this study in the accuracy of the provision for these regions. On the other hand the impact of the differences in geometry and relations of the organs between the animals and, thus, the scaling to human orders of magnitude as a systematic source of error has more influence on the accuracy of the dose calculation. Finally, there is a rather low fraction of activity that enters the gut system as seen in the tables and fits below. Obviously, the initially high fraction of injected activity in the liver is eliminated mainly by the blood and due to the fact that the kinetics in the hepatobiliary pathway is slower than that of the elimination through blood and the renal clearance. So, even being a relatively radiation sensible tissue, the overall contribution of the gut region to the effective dose is rather low as shown in table S4.

Table S1: %ID values of the first subject (FACHpig1, weight 15kg) after i.v. injection of 190,9 MBq [^{18}F]FACH followed by sequential PET/CT imaging according time schedule in the very first left column.

Organ/Compartment		Brain			Small Intestine			Stomach			ULI			Myocard			Kidneys			Liver			Gallbladder		
Modell		Animal		Human	Animal		Human	Animal		Human	Animal		Human	Animal		Human	Animal		Human	Animal		Human	Animal		Human
Organmass [g]		83		1420	395		677	356		158	344		387	266		316	140		299	558		1910			56
Fractions		%ID per organ	%ID per g	%ID per organ	%ID per organ	%ID per g	%ID per organ	%ID per organ	%ID per g	%ID per organ	%ID per organ	%ID per g	%ID per organ	%ID per organ	%ID per g	%ID per organ	%ID per organ	%ID per g	%ID per organ	%ID per organ	%ID per g	%ID per organ	%ID per organ	%ID per g	%ID per organ
$Time_{animal}$ [h]	$Time_{human}$ [h]																								
0.00	0.00	0.2	0.002	0.7	1.2	0.003	0.4	0.8	0.002	0.08	3.3	0.010	0.8	1.9	0.007	0.47	10.0	0.072	4.4	28.7	0.051	20.0	not identifiable		
0.18	0.27	0.1	0.002	0.5	1.1	0.003	0.4	0.7	0.002	0.07	3.5	0.010	0.8	1.7	0.006	0.42	6.7	0.048	2.9	20.6	0.037	14.3			
0.37	0.55	0.2	0.002	0.6	1.2	0.003	0.4	0.7	0.002	0.06	3.4	0.010	0.8	1.4	0.005	0.34	5.6	0.040	2.4	16.5	0.029	11.5			
0.55	0.82	0.2	0.002	0.6	2.2	0.006	0.8	1.0	0.003	0.09	2.0	0.006	0.5	1.2	0.004	0.28	5.1	0.036	2.2	16.0	0.029	11.1			
0.73	1.09	0.2	0.003	0.7	2.8	0.007	1.0	1.1	0.003	0.10	1.5	0.004	0.4	1.1	0.004	0.26	2.8	0.020	1.2	15.4	0.028	10.7			
0.97	1.44	0.2	0.003	0.8	1.9	0.005	0.7	0.9	0.003	0.08	1.9	0.006	0.4	1.0	0.004	0.25	2.4	0.017	1.1	13.8	0.025	9.6			
1.20	1.79	0.2	0.003	0.9	2.2	0.006	0.8	1.2	0.003	0.11	1.5	0.004	0.3	0.9	0.004	0.23	1.5	0.011	0.7	13.1	0.023	9.1			
1.90	2.83	0.2	0.003	0.8	3.8	0.010	1.3	1.7	0.005	0.15	1.5	0.004	0.3	0.8	0.003	0.19	1.2	0.008	0.5	6.7	0.012	4.6			
2.72	4.04	0.2	0.003	0.8	3.0	0.008	1.1	3.8	0.011	0.34	2.3	0.007	0.5	0.7	0.003	0.18	1.1	0.008	0.5	7.4	0.013	5.2			
3.63	5.41	0.2	0.003	0.8	3.7	0.009	1.3	3.4	0.010	0.31	2.7	0.008	0.6	0.7	0.003	0.17	0.9	0.006	0.4	6.3	0.011	4.4			

• • •

• • •

Organ/Compartment		Lungs			Pancreas			Red Marrow			Spleen			Bone			Thyroid			Urinary Bladder			Reminder of Body		
Modell		Animal		Human	Animal		Human	Animal		Human	Animal		Human	Animal		Human	Animal		Human	Animal		Human	Animal		Human
Organmass [g]		595		1000	48		94	406		1120	1		183	1000		4000	9		21	76		211	10723		61849
Fractions		%ID per organ	%ID per g	%ID per organ	%ID per organ	%ID per g	%ID per organ	%ID per organ	%ID per g	%ID per organ	%ID per organ	%ID per g	%ID per organ	%ID per organ	%ID per g	%ID per organ	%ID per organ	%ID per g	%ID per organ	%ID per organ	%ID per g	%ID per organ	%ID per organ	%ID per g	%ID per organ
$Time_{animal}$ [h]	$Time_{human}$ [h]																								
0.00	0.00	3.0	0.005	1.0	0.5	0.011	0.2	2.0	0.005	1.1	0.0	0.003	0.1	4.1	0.004	3.33	0.0	0.002	0.007	0.3	0.004	0.2	43.9	0.004	51.57
0.18	0.27	2.4	0.004	0.8	1.2	0.025	0.5	2.9	0.007	1.6	0.0	0.003	0.1	6.1	0.006	4.93	0.0	0.003	0.011	1.6	0.021	0.9	51.4	0.005	60.29
0.37	0.55	2.1	0.004	0.7	6.1	0.128	2.4	3.4	0.008	1.9	0.0	0.002	0.1	7.1	0.007	5.80	0.0	0.002	0.010	4.4	0.058	2.5	47.9	0.004	56.19
0.55	0.82	1.7	0.003	0.6	5.7	0.118	2.3	3.6	0.009	2.0	0.0	0.003	0.1	7.7	0.008	6.29	0.0	0.003	0.012	5.8	0.077	3.3	47.8	0.004	56.14
0.73	1.09	1.7	0.003	0.6	6.0	0.125	2.4	3.6	0.009	2.0	0.0	0.003	0.1	7.8	0.008	6.38	0.0	0.003	0.013	8.7	0.115	4.9	47.1	0.004	55.30
0.97	1.44	1.6	0.003	0.6	3.9	0.082	1.6	4.0	0.010	2.3	0.0	0.003	0.1	8.4	0.008	6.83	0.0	0.003	0.014	9.8	0.129	5.6	49.9	0.005	58.60
1.20	1.79	1.7	0.003	0.6	3.6	0.074	1.4	3.8	0.009	2.1	0.0	0.003	0.1	8.3	0.008	6.72	0.0	0.003	0.014	9.3	0.122	5.2	52.7	0.005	61.83
1.90	2.83	2.2	0.004	0.7	4.2	0.087	1.7	3.4	0.008	1.9	0.0	0.000	0.0	6.8	0.007	5.52	0.0	0.003	0.012	9.8	0.129	5.6	57.8	0.005	67.80
2.72	4.04	2.1	0.004	0.7	4.3	0.089	1.7	3.2	0.008	1.8	0.0	0.001	0.0	7.1	0.007	5.79	0.0	0.002	0.009	10.8	0.141	6.1	54.0	0.005	63.39
3.63	5.41	2.2	0.004	0.8	3.5	0.074	1.4	3.1	0.008	1.7	0.0	0.001	0.0	6.8	0.007	5.54	0.0	0.002	0.008	9.6	0.127	5.4	56.8	0.005	66.67

Table S2: %ID values of the second subject (FACHpig2. weight 13.5kg) after i.v. injection of 94.2 MBq [^{18}F]FACH followed by sequential PET/CT imaging according time schedule in the very first left column.

**Organ/Compartment**		**Brain**			**Small Intestine**			**Stomach**			**ULI**			**Myocard**			**Kidneys**			**Liver**			**Gallbladder**		
**Modell**		**Animal**		**Human**	**Animal**		**Human**	**Animal**		**Human**	**Animal**		**Human**	**Animal**		**Human**	**Animal**		**Human**	**Animal**		**Human**	**Animal**		**Human**
**Organmass [g]**		43		1420	646		677	557		158	1092		387	173		316	154		299	468		1910	18		56
**Fractions**		**%ID per**		**%ID per**	**%ID per**		**%ID per**	**%ID per**		**%ID per**	**%ID per**		**%ID per**	**%ID per**		**%ID per**	**%ID per**		**%ID per**	**%ID per**		**%ID per**	**%ID per**		**%ID per**
**Time$_{animal}$ [h]**	**Time$_{human}$ [h]**	**organ**	**g**	**organ**	**organ**	**g**	**organ**	**organ**	**g**	**organ**	**organ**	**g**	**organ**	**organ**	**g**	**organ**	**organ**	**g**	**organ**	**organ**	**g**	**organ**	**organ**	**g**	**organ**
0.00	0.00	0.1	0.002	0.4	4.3	0.007	0.8	2.2	0.004	0.1	3.7	0.003	0.2	1.4	0.008	0.5	8.5	0.055	3.0	33.1	0.071	24.8	0.8	0.05	0.48
0.18	0.27	0.0	0.001	0.3	7.3	0.011	1.4	1.7	0.003	0.1	4.3	0.004	0.3	1.2	0.007	0.4	8.9	0.058	3.2	22.7	0.048	16.9	0.7	0.04	0.40
0.37	0.55	0.1	0.001	0.4	9.7	0.015	1.9	1.4	0.003	0.1	3.5	0.003	0.2	1.0	0.006	0.3	6.9	0.045	2.4	15.8	0.034	11.8	0.8	0.05	0.47
0.55	0.82	0.1	0.002	0.4	12.5	0.019	2.4	1.2	0.002	0.1	2.9	0.003	0.2	0.8	0.005	0.3	5.2	0.034	1.9	11.5	0.024	8.6	1.7	0.09	0.96
0.73	1.09	0.1	0.002	0.4	17.6	0.027	3.4	1.2	0.002	0.1	2.4	0.002	0.2	0.7	0.004	0.2	3.1	0.020	1.1	8.2	0.017	6.1	2.3	0.13	1.30
0.97	1.44	0.1	0.002	0.5	19.8	0.031	3.8	1.2	0.002	0.1	2.2	0.002	0.1	0.6	0.003	0.2	2.4	0.016	0.9	6.3	0.014	4.7	2.3	0.13	1.34
1.20	1.79	0.1	0.002	0.5	21.3	0.033	4.1	1.1	0.002	0.1	2.1	0.002	0.1	0.5	0.003	0.2	1.8	0.012	0.6	5.3	0.011	4.0	2.2	0.13	1.29
1.90	2.83	0.1	0.002	0.6	14.0	0.022	2.7	1.0	0.002	0.0	2.1	0.002	0.1	0.3	0.002	0.1	0.9	0.006	0.3	3.8	0.008	2.9	1.6	0.09	0.91
2.72	4.04	0.1	0.002	0.6	13.3	0.021	2.6	1.2	0.002	0.1	2.2	0.002	0.1	0.3	0.002	0.1	0.5	0.003	0.2	3.0	0.006	2.3	1.2	0.07	0.70
3.63	5.41	0.1	0.002	0.6	12.1	0.019	2.3	1.0	0.002	0.1	3.5	0.003	0.2	0.3	0.002	0.1	0.5	0.003	0.2	2.3	0.005	1.7	1.2	0.07	0.68

• • •

• • •

**Organ/Compartment**		**Lungs**			**Pancreas**			**Red Marrow**			**Spleen**			**Bone**			**Thyroid**			**Urinary Bladder**			**Reminder of Body**		
**Modell**		**Animal**		**Human**	**Animal**		**Human**	**Animal**		**Human**	**Animal**		**Human**	**Animal**		**Human**	**Animal**		**Human**	**Animal**		**Human**	**Animal**		**Human**
**Organmass [g]**		481		1000	42		94	176		1120	46		183	0		4000	7		21	258		211	9357		65828
**Fractions**		**%ID per**		**%ID per**	**%ID per**		**%ID per**	**%ID per**		**%ID per**	**%ID per**		**%ID per**	**%ID per**		**%ID per**	**%ID per**		**%ID per**	**%ID per**		**%ID per**	**%ID per**		**%ID per**
**Time$_{animal}$ [h]**	**Time$_{human}$ [h]**	**organ**	**g**	**organ**	**organ**	**g**	**organ**	**organ**	**g**	**organ**	**organ**	**g**	**organ**	**organ**	**g**	**organ**	**organ**	**g**	**organ**	**organ**	**g**	**organ**	**organ**	**g**	**organ**
0.00	0.00	3.0	0.006	1.1	0.3	0.006	0.1	1.4	0.008	1.6	0.3	0.006	0.2	5.7	0.004	3.0	0.03	0.004	0.016	0.5	0.002	0.1	40.4	0.004	52.10
0.18	0.27	2.4	0.005	0.9	0.9	0.022	0.4	2.3	0.013	2.7	0.2	0.004	0.1	8.9	0.006	4.7	0.03	0.004	0.016	2.9	0.011	0.4	44.5	0.005	57.39
0.37	0.55	2.1	0.004	0.8	2.6	0.061	1.0	3.0	0.017	3.5	0.2	0.004	0.1	11.9	0.008	6.2	0.02	0.004	0.013	6.2	0.024	0.9	46.6	0.005	60.11
0.55	0.82	1.9	0.004	0.7	4.2	0.100	1.7	3.9	0.022	4.5	0.2	0.004	0.1	15.3	0.011	8.0	0.02	0.003	0.011	9.1	0.035	1.4	44.9	0.005	57.91
0.73	1.09	1.7	0.004	0.7	1.1	0.026	0.4	4.5	0.026	5.2	0.2	0.003	0.1	18.5	0.013	9.7	0.02	0.003	0.010	11.1	0.043	1.7	45.9	0.005	59.11
0.97	1.44	1.6	0.003	0.6	0.8	0.019	0.3	4.9	0.028	5.8	0.1	0.003	0.1	21.7	0.015	11.3	0.01	0.002	0.008	12.6	0.049	1.9	44.9	0.005	57.91
1.20	1.79	1.6	0.003	0.6	0.7	0.018	0.3	5.2	0.030	6.1	0.1	0.003	0.1	24.9	0.018	13.0	0.01	0.002	0.006	13.5	0.052	2.0	44.4	0.005	57.27
1.90	2.83	1.9	0.004	0.7	0.6	0.014	0.2	5.1	0.029	6.0	0.2	0.003	0.1	14.7	0.011	7.7	0.01	0.001	0.005	15.7	0.061	2.3	52.8	0.006	68.09
2.72	4.04	1.9	0.004	0.7	0.5	0.011	0.2	4.9	0.028	5.7	0.2	0.004	0.1	14.7	0.011	7.7	0.01	0.001	0.004	17.9	0.069	2.7	52.8	0.006	68.06
3.63	5.41	2.1	0.004	0.8	0.3	0.007	0.1	4.4	0.025	5.1	0.2	0.004	0.1	14.0	0.010	7.3	0.01	0.001	0.004	18.1	0.070	2.7	53.8	0.006	69.33

Table S3: %ID values of the third subject (FACHpig3. weight 18kg) after i.v. injection of 185.0 MBq [^{18}F]FACH followed by sequential PET/CT imaging according time schedule in the very first left column.

Organ/Compartment		Brain			Small Intestine			Stomach			ULI			Myocard			Kidneys			Liver			Gallbladder		
Modell		Animal		Human	Animal		Human	Animal		Human	Animal		Human	Animal		Human	Animal		Human	Animal		Human	Animal		Human
Organmass [g]		53		1420	0		677	863		158	1034		387	184		316	144		299	529		1910	25		56
Fractions		%ID per		%ID per	%ID per		%ID per	%ID per		%ID per	%ID per		%ID per	%ID per		%ID per	%ID per		%ID per	%ID per		%ID per	%ID per		%ID per
$Time_{animal}$ [h]	$Time_{human}$ [h]	organ	g	organ	organ	g	organ	organ	g	organ	organ	g	organ	organ	g	organ	organ	g	organ	organ	g	organ	organ	g	organ
0.00	0.00	0.1	0.001	0.5	3.0	0.004	0.7	1.0	0.001	0.04	3.4	0.003	0.3	1.4	0.008	0.6	10.9	0.076	5.5	39.0	0.074	34.4	1.1	0.05	0.62
0.18	0.27	0.0	0.001	0.3	3.7	0.005	0.8	0.7	0.001	0.03	3.8	0.004	0.3	1.0	0.006	0.4	9.0	0.062	4.5	27.3	0.052	24.1	2.7	0.11	1.47
0.37	0.55	0.1	0.001	0.3	8.6	0.012	1.9	0.6	0.001	0.03	7.5	0.007	0.7	0.9	0.005	0.4	6.6	0.046	3.4	17.1	0.032	15.1	3.6	0.15	1.98
0.55	0.82	0.1	0.001	0.4	13.9	0.019	3.1	0.6	0.001	0.03	11.2	0.011	1.0	0.8	0.004	0.3	4.3	0.030	2.2	12.3	0.023	10.8	3.7	0.15	2.01
0.73	1.09	0.1	0.001	0.4	18.0	0.024	4.0	0.7	0.001	0.03	15.7	0.015	1.4	0.6	0.003	0.3	2.5	0.017	1.3	9.1	0.017	8.0	3.4	0.14	1.88
0.97	1.44	0.1	0.001	0.5	20.1	0.027	4.5	0.8	0.001	0.03	17.5	0.017	1.6	0.5	0.003	0.2	2.0	0.014	1.0	7.3	0.014	6.4	4.0	0.16	2.16
1.20	1.79	0.1	0.001	0.5	20.3	0.028	4.6	0.8	0.001	0.04	17.2	0.017	1.6	0.5	0.002	0.2	1.5	0.010	0.7	5.8	0.011	5.1	3.5	0.14	1.88
1.90	2.83	0.1	0.002	0.6	18.6	0.025	4.2	0.9	0.001	0.04	19.1	0.019	1.7	0.0	0.000	0.0	0.8	0.005	0.4	4.2	0.008	3.7	3.5	0.14	1.88
2.72	4.04	0.1	0.002	0.7	20.9	0.028	4.7	0.8	0.001	0.04	17.6	0.017	1.6	0.0	0.000	0.0	0.4	0.003	0.2	3.0	0.006	2.7	3.4	0.14	1.87
3.63	5.41	0.1	0.002	0.5	19.5	0.027	4.4	0.8	0.001	0.04	15.1	0.015	1.4	0.0	0.000	0.0	0.7	0.005	0.4	3.2	0.006	2.8	1.9	0.08	1.05

• • •

• • •

Organ/Compartment		Lungs			Pancreas			Red Marrow			Spleen			Bone			Thyroid			Urinary Bladder			Reminder of Body		
Modell		Animal		Human	Animal		Human	Animal		Human	Animal		Human	Animal		Human	Animal		Human	Animal		Human	Animal		Human
Organmass [g]		393		1000	42		94	349		1120	0		183	1061		4000	7		21	72		211	13269		61849
Fractions		%ID per		%ID per	%ID per		%ID per	%ID per		%ID per	%ID per		%ID per	%ID per		%ID per	%ID per		%ID per	%ID per		%ID per	%ID per		%ID per
$Time_{animal}$ [h]	$Time_{human}$ [h]	organ	g	organ	organ	g	organ	organ	g	organ	organ	g	organ	organ	g	organ	organ	g	organ	organ	g	organ	organ	g	organ
0.00	0.00	3.0	0.008	1.9	0.05	0.001	0.03	1.7	0.005	1.3				4.1	0.004	3.751	0.01	0.002	0.008	0.2	0.003	0.2	34.0	0.003	38.75
0.18	0.27	1.9	0.005	1.2	0.05	0.001	0.03	1.7	0.005	1.4				4.3	0.004	3.960	0.01	0.002	0.009	2.2	0.030	1.6	45.4	0.003	51.69
0.37	0.55	1.7	0.004	1.0	0.04	0.001	0.02	2.5	0.007	2.0				6.0	0.006	5.548	0.01	0.002	0.010	6.2	0.087	4.5	47.1	0.004	53.59
0.55	0.82	1.3	0.003	0.8	0.03	0.001	0.02	3.2	0.009	2.5				7.5	0.007	6.889	0.02	0.002	0.011	10.0	0.139	7.1	45.1	0.003	51.39
0.73	1.09	1.2	0.003	0.7	0.03	0.001	0.01	3.6	0.010	2.9	not identifiable			8.3	0.008	7.642	0.02	0.002	0.011	12.7	0.176	9.1	42.1	0.003	47.90
0.97	1.44	1.0	0.003	0.6	0.01	0.000	0.01	3.9	0.011	3.1				8.9	0.008	8.225	0.02	0.002	0.011	13.5	0.188	9.7	40.5	0.003	46.16
1.20	1.79	1.0	0.002	0.6	0.02	0.000	0.01	4.2	0.012	3.3				9.5	0.009	8.790	0.02	0.002	0.011	13.4	0.187	9.6	42.6	0.003	48.52
1.90	2.83	0.7	0.002	0.4	0.06	0.001	0.03	4.4	0.013	3.5				11.0	0.010	10.088	0.03	0.004	0.020	10.0	0.139	7.2	45.2	0.003	51.49
2.72	4.04	0.6	0.001	0.3	0.01	0.000	0.00	3.7	0.011	2.9				10.5	0.010	9.623	0.03	0.005	0.025	6.6	0.092	4.7	53.2	0.004	60.51
3.63	5.41	0.9	0.002	0.6	0.02	0.000	0.01	3.2	0.009	2.5				8.9	0.008	8.215	0.01	0.002	0.009	9.4	0.131	6.8	55.7	0.004	63.42

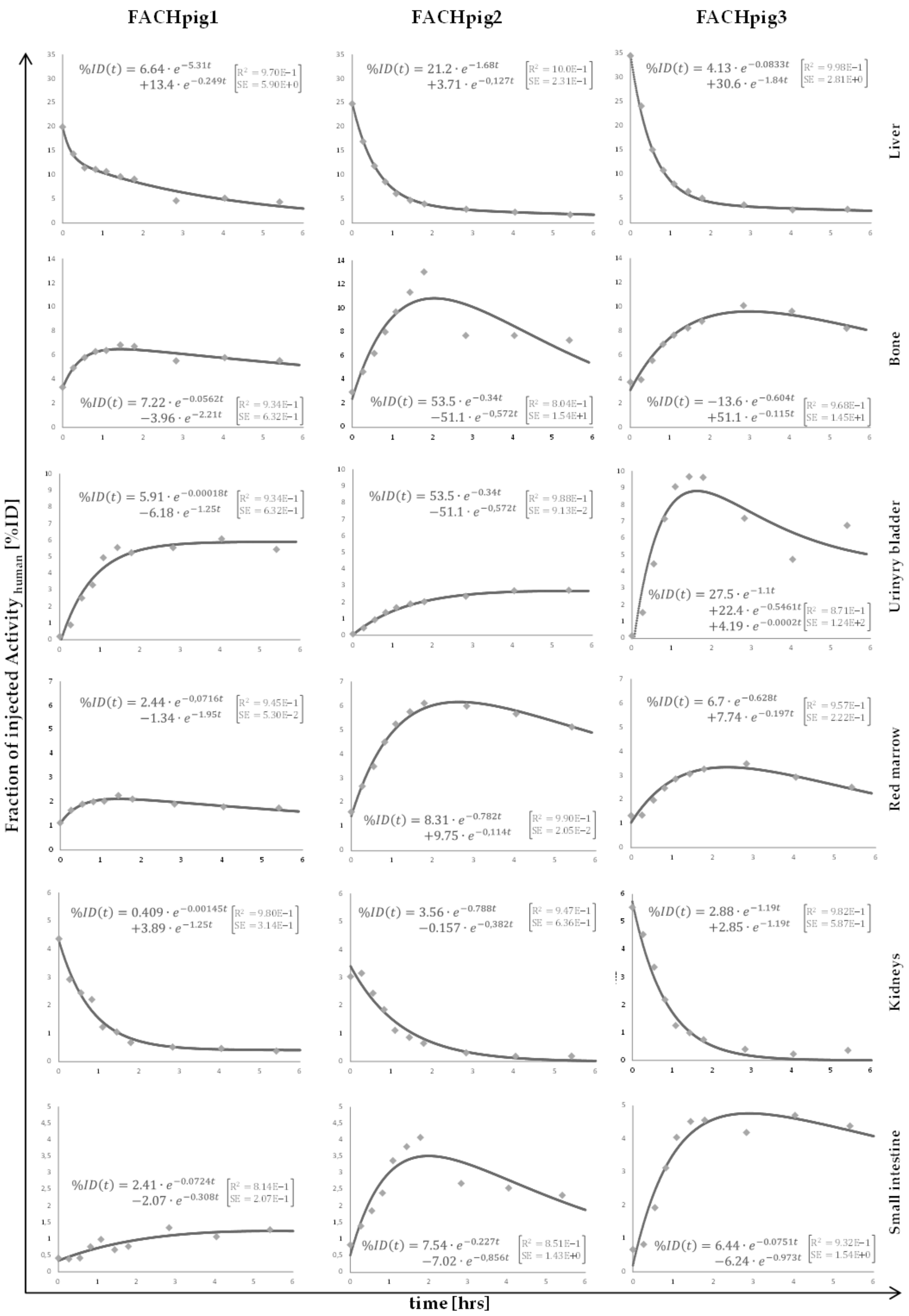

Figure S1: Mono-, bi- or tri-exponential fits of the TIACs for all subjects (columns) and organs and systems of organs (rows) that are identifiable in the structural CT data and/or by considerable activity uptake/concentration in the PET data along with the particular fit functions and fit goodness parameters (R-squared and squared error), sorted by %ID in descending order.

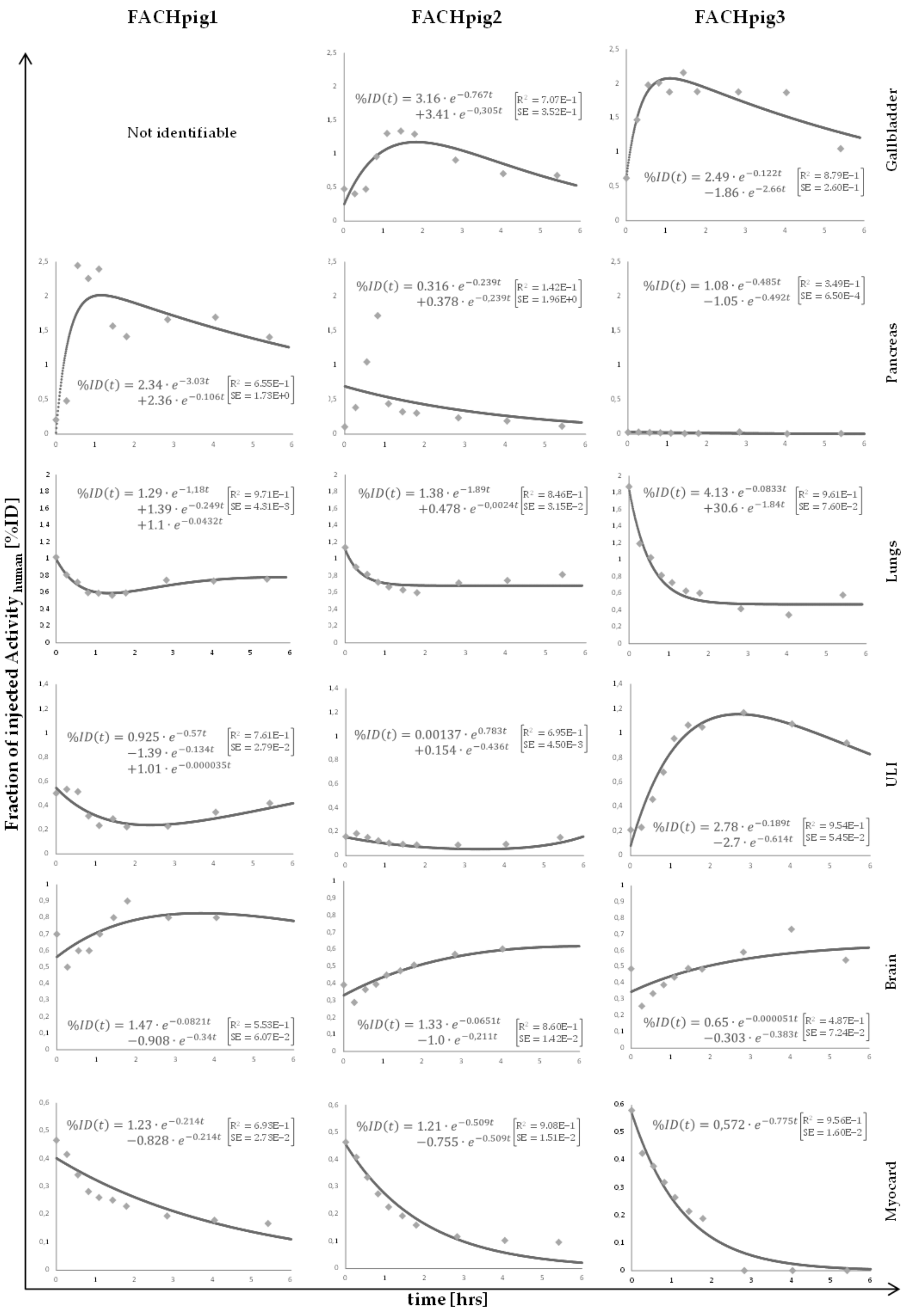

Figure S2: Mono-, bi- or tri-exponential fits of the TIACs for all subjects (columns) and organs and systems of organs (rows) that are identifiable in the structural CT data and/or by considerable activity uptake/concentration in the PET data along with the particular fit functions and fit goodness parameters (R-squared and squared error). (Figure S1 continued)

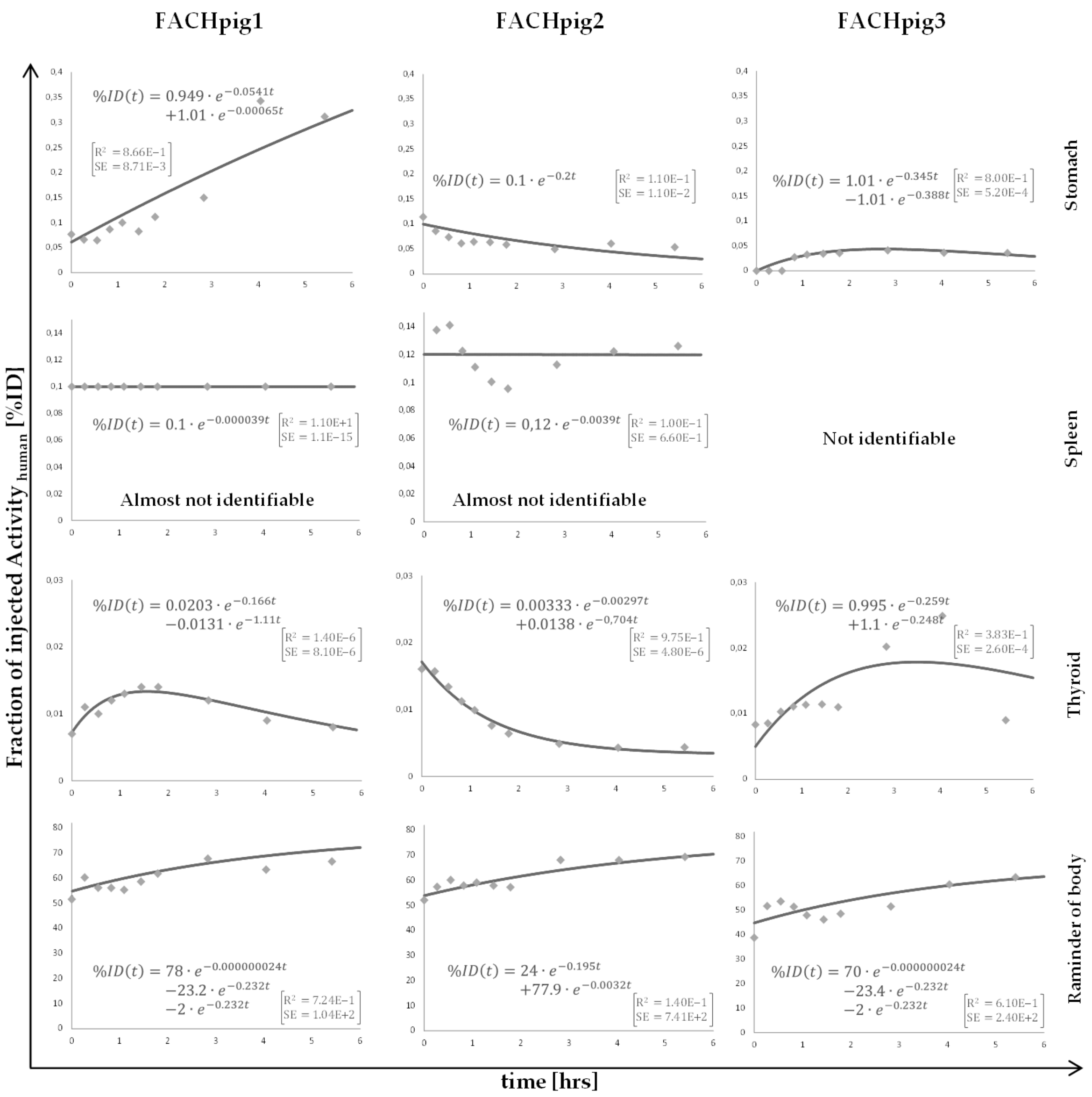

Figure S3: Mono-, bi- or tri-exponential fits of the TIACs for all subjects (columns) and organs and systems of organs (rows) that are identifiable in the structural CT data and/or by considerable activity uptake/concentration in the PET data along with the particular fit functions and fit goodness parameters (R-squared and squared error). (Figure S2 continued)

Table S4: Detailed results of the dose calculation for the three animals: organ equivalent doses and effective dose contributions involving the tissue risk factor w_T of ICRP 103 for weighting the organs and organ systems for their radiation sensibility

**Organ/ Organsystem**	**Organ equivalent dose H_T [mSv/MBq]**			**Effective dose contribution $H_T \cdot w_T$ [mSv/MBq]**		
	FACHpig1	***FACHpig2***	***FACHpig3***	***FACHpig1***	***FACHpig2***	***FACHpig3***
Adrenals	1.36E-02	1.44E-02	1.47E-02	1.26E-04	1.33E-04	1.35E-04
Brain	5.61E-03	4.35E-03	4.10E-03	5.61E-05	4.35E-05	4.10E-05
Esophagus	1.04E-02	8.58E-03	8.11E-03	4.16E-04	3.43E-04	3.24E-04
Eyes	8.81E-03	6.29E-03	5.63E-03	0.00E+00	0.00E+00	0.00E+00
Gallbladder Wall	1.22E-02	5.18E-02	8.72E-02	1.12E-04	4.78E-04	8.05E-04
Left colon	1.60E-02	1.46E-02	1.04E-02	7.77E-04	7.07E-04	5.06E-04
Small Intestine	1.88E-02	2.67E-02	9.84E-03	1.74E-04	2.47E-04	9.09E-05
Stomach Wall	1.43E-02	1.01E-02	8.32E-03	1.71E-03	1.22E-03	9.98E-04
Right colon	1.93E-02	3.25E-02	2.01E-02	9.34E-04	1.58E-03	9.75E-04
Rectum	1.50E-02	1.38E-02	1.27E-02	3.44E-04	3.17E-04	2.92E-04
Heart Wall	1.03E-02	9.21E-03	8.86E-03	9.47E-05	8.50E-05	8.18E-05
Kidneys	2.67E-02	2.38E-02	2.81E-02	2.47E-04	2.20E-04	2.59E-04
Liver	8.05E-03	2.77E-02	3.42E-02	3.22E-04	1.11E-03	1.37E-03
Lungs	8.05E-03	7.96E-03	7.62E-03	9.67E-04	9.55E-04	9.15E-04
Pancreas	6.08E-02	2.28E-02	7.85E-03	5.61E-04	2.11E-04	7.24E-05
Prostate	1.59E-02	1.02E-02	1.29E-02	7.36E-05	4.73E-05	5.95E-05
Salivary Glands	1.03E-02	7.07E-03	6.33E-03	1.03E-04	7.07E-05	6.33E-05
Red Marrow	1.32E-02	1.64E-02	1.22E-02	1.58E-03	1.97E-03	1.47E-03
Osteogenic Cells	2.00E-02	2.34E-02	2.03E-02	2.00E-04	2.34E-04	2.03E-04
Spleen	1.03E-02	8.88E-03	8.05E-03	9.48E-05	8.20E-05	7.43E-05
Testes	1.10E-02	7.08E-03	7.26E-03	4.40E-04	2.83E-04	2.90E-04
Thymus	1.03E-02	7.66E-03	7.04E-03	9.49E-05	7.07E-05	6.50E-05
Thyroid	8.02E-03	6.30E-03	6.93E-03	3.21E-04	2.52E-04	2.77E-04
Urinary Bladder Wall	6.86E-02	3.12E-02	8.80E-02	2.74E-03	1.25E-03	3.52E-03
Total Body	1.15E-02	9.14E-03	8.63E-03	0.00E+00	0.00E+00	0.00E+00
			**Effective dose:**	1.25E-02	1.19E-02	1.29E-02
